# Nuclear bodies: a gene expression collection for our time

**DOI:** 10.1080/19491034.2024.2339580

**Published:** 2024-04-14

**Authors:** Thoru Pederson

**Affiliations:** Department of Biochemistry and Molecular Biotechnology, University of Massachusetts Chan Medical School, Worcester, MA, USA

Next year marks the 100^th^ anniversary of Edmund B. Wilson’s iconic book ‘The Cell in Development and Heredity’. Its actual centennial would have been 1996 (the first edition), but it was the third (1925) that caught on. With penetrating erudition Wilson presented the nucleus in a sweeping landscape of topics such as mitosis, meiosis, fertilization, sex determination and embryonic development. I am currently co-authoring a historical reflection on the book but for this editorial it, and its author, stand deservedly as the point of departure.

The nucleus at interphase was a lesser focus in Wilson’s time, both as knowledge and progress. The revealed chromosomal basis of heredity summoned images of coiled chromosomes in dividing cells but their actions in between were not even remotely understood. In the latter part of the 19^th^ century, and the first quarter of the 20^th^, one finds little curiosity on what genes really do, and whether their residence in the cell nucleus is autonomous or collaborates with other elements in that organelle, such ‘other elements’ then only dimly known.

The first case of an apparent non-DNA, non-chromosomal entity in the nucleus (as it was perceived then but of course we now know otherwise) was the nucleolus. Its discovery is often attributed to a discredited 18^th^ century observation but the definitive reports came later [1,2]. The second case was a body described by Ramon y Cajal, using his staining method that was the basis for the work for which he shared the 1906 Nobel Prize. This body later got named the ‘coiled body’ and we owe an epistemological debt to Joe Gall for his successful campaign to get it renamed for its founder [3].

My own intrigue with nuclear bodies began with the nucleolus. In a college cytochemistry lab course it jumped out at me after applying certain dyes- almost like a siren beckoning. Over my career I have pursued the nucleolus and other nuclear bodies and last year suggested that *Nucleus* organize this Collection, given how impactfully this field has been progressing. I was delighted that each author I invited accepted, and with enthusiasm.

Rohit Pappu and colleagues [4] provide an insightful and comprehensive treatment of ‘The Nucleolus 2024’. Their article captures important recent developments and addresses the liquid-liquid phase separation dimensions of this intriguing organelle in as lucid a manner as anything published before. They also include new data of their own, just being published, that defines a new way of thinking about the tripartite signature of the nucleolus and the physical chemistry that underlies each component.

Eugene Makeyev and Sui Huang in their article [5] bring us up to date on a fascinating body that is situated adjacent to the nucleolus in many cells, the perinucleolar compartment (PNC). I confess a particular connection to Sui Huang because in a collaboration we saw the PNC very early (1991) in a collaborative study, with David Spector. Another group reported it a year later but, to her credit, Huang pushed on. With increasing evidence for a correlation between the presence of PNCs in tumor cell lines and their metastatic potential, she undertook studies with the National Cancer Institute. The result is a lead candidate drug, metarrestin, that may be the first to emerge in the history of cancer chemotherapy that inhibits cancer progression (metastasis) as opposed to being preferentially cytostatic for dividing tumor cells.

David Staněk [6] has provided a comprehensive and insightful account of Cajal bodies, both as to the early eras and current trends. His mastery of the subject is evident and allows one to form a picture of this organelle that expands the key issue of inter-nuclear body traffic. Recent work has suggested that the nucleolus governs the Cajal body [7] (and maybe vice-versa). Staněk’s article leaves the door open for a future in which we may come to a deeper understanding of how all these nuclear bodies interact.

The article by Majdouline Abou-Ghali and Valerie Lallemand-Breitenbach on PML bodies [8] complements the one on the PNC in that these bodies were also discovered years ago but acquired a functional connection only later, when their presence was noted to be correlated with cancer cells and, in particular, was related to an oncogenic fusion of a PML body protein gene with that encoding the retinoic acid A receptor. The authors engagingly describe this evolution as well as the particular connection of PML bodies to hematological cancers. As with Sui Huang’s metarrestin, promising clinical results are coming from drugs based on PML body basic science.

The Histone Locus Body is something of an orphan and yet has been the basis for a significant enrichment of our understanding of the full repertoire of nuclear bodies. Unlike other nuclear bodies, it is stationed on the genome, at a particular site. This neither dismisses it from the atlas of Nuclear Bodies but makes it even more intriguing. Geisler, Kemp and Duronio have made it so not only in their own work but in their engaging article [9]. Here we see a body whose genomic residence arises from the activity of certain genes. This article is a perfect complement to this Collection for this reason.

In closing, we in this field may feel like the famous line in *Hamlet*: ‘There are more things in heaven and earth, Horatio, than are dreamt of in your philosophy’. And I would agree. So, we must keep an open mind and dare to imagine discoveries yet to come in this field. In his Nobel Prize acceptance lecture (1975) the pioneer cell biologist Albert Claude said: ‘We have entered the cell, the Mansion of our birth, and started the inventory of our acquired wealth’. This was half a century ago and it still rings true today.

I thank all the authors of this Collection. In their laboratories they have collectively made a mark in this field. They have now also contributed to a record of the current era that will stand for years as the definitive treatment of the subject that it is.
